# Rapid and Direct Detection of the Stubby Root Nematode, *Paratrichodorus allius,* from Soil DNA Extracts Using Recombinase Polymerase Amplification Assay

**DOI:** 10.3390/ijms251910371

**Published:** 2024-09-26

**Authors:** Mankanwal Goraya, Guiping Yan

**Affiliations:** Department of Plant Pathology, North Dakota State University, Fargo, ND 58108, USA; mfg5907@psu.edu

**Keywords:** stubby root nematode, diagnostics, ITS-rDNA, real-time RPA, species-specific assay

## Abstract

The stubby root nematode, *Paratrichodorus allius,* is one of the most important plant-parasitic nematodes. Besides root feeding, *P. allius* also transmits the *Tobacco rattle virus* in potatoes, which causes corky ringspot disease. Rapid detection of *P. allius* is key for efficient management. This study was conducted to develop a real-time recombinase polymerase amplification (RPA) assay that is capable of detecting *P. allius* directly in DNA extracts from soil using a simple portable device in real time. A fluorophore-attached probe was designed to target the internal transcribed spacer (ITS)-rDNA of *P. allius* and was used along with primers designed previously. The real-time RPA assay had the ability to detect *P. allius* DNA extracted directly from infested soil with a sensitivity of one-sixteenth portion of a single nematode. This RPA assay was specific, as it did not produce positive signals from non-target nematodes tested. The real-time RPA was found to be rapid as it could even detect *P. allius* in as little as 7 min. Testing with 15 field soil samples validated the RPA assay developed in this study. This is the first report of *P. allius* detection directly from soil DNA using real-time RPA and is the fastest method for *P. allius* detection in soil to date.

## 1. Introduction

Plant-parasitic nematodes are important pests that impact crop productivity and reduce crop yield. The symptoms of nematode diseases often resemble those caused by other plant pathogens and common soil ailments [1]. The stubby root nematodes are polyphagous plant-parasitic nematodes [1] that damage many crops, including potato, sugar beet, and other Poaceae family crops. Stubby root nematodes are ectoparasites that feed by puncturing the wall of the root tip cell with their onchiostyle and ingesting the cell content after salivation [1,2,3,4,5,6]. They belong to the Trichodoridae family [6], with the *Trichodorus* and *Paratrichodorus* genera being widely distributed globally [7,8]. Certain species of *Paratrichodorus*, besides causing direct yield loss, are also responsible for transmitting viruses to the plants; they include *Tobacco rattle virus*, *Pea-early browning virus*, and *Pepper ringspot virus* [5,6,7]. The presence of stubby root nematodes in the rhizosphere of affected plants cannot be determined by specific symptoms shown by their aboveground parts [8]. Nematological analysis of soil supplemented by efficient diagnostic methods is necessary to verify the presence of these nematodes in the soil.

*Paratrichodorus allius* is one of the important stubby root nematodes that cause dual damage to potato crops by feeding on the roots [9] and transmitting the *Tobacco rattle virus* [10]. This virus is responsible for corky ringspot disease in potatoes [11], a disease that adversely affects the quality of the tubers, resulting in the rejection of potato lots by the potato industry [12]. *Paratrichodorus allius* has been reported in multiple states across the United States, including Oregon [13], Michigan [14], Ohio [15], North Dakota [16], Minnesota [17], Georgia [18], North Carolina, South Carolina [19], Florida [1], and other states in the Pacific Midwest region [20]. Because of its widespread distribution and the severe damage that it causes to potato crops, effective management of *P. allius* is crucial.

It is essential to identify *P. allius* to design effective strategies for managing this nematode pest. There are multiple methods for detecting *Paratrichodorus* spp. First, species-level differentiation can be conducted using morphometric measurements; however, species differentiation using morphological and morphometric analysis is challenging and requires high taxonomic skills [21]. Minor characteristics are often confused among closely related species, for example, *P. teres* and *P. allius* have subtle differences based on vaginal shape [22]. In soil populations with more than one species of *Paratrichodorus*, identification could be highly difficult. In order to improve detection, molecular diagnostic methods exploring ribosomal DNA (rDNA) and internal transcribed spacers (ITS-1 and ITS-2) regions have been developed to differentiate species. For molecular characterizations of stubby root nematodes, many studies have been conducted using DNA sequencing, conventional polymerase chain reaction (PCR), PCR restriction fragment length polymorphism (RFLP), quantitative PCR (qPCR), multiplex PCR, and droplet digital PCR (ddPCR) [22,23,24,25,26,27,28,29]. Although the molecular techniques developed so far for the *Paratrichodorus* spp., such as DNA sequencing and various forms of PCR, provide precise identification at the species level, all these techniques require lab-oriented environments, provision of a different set of temperature settings, as well as a considerable amount of time for the identification of nematode species. Therefore, there is a need for a technique that is easy to operate at a constant temperature with reduced time of detection and that can operate in diverse environments and not be limited to laboratory settings.

Recombinase polymerase amplification (RPA) assay is one of the isothermal techniques that is easy to operate. RPA assays have been developed to identify plant-parasitic nematodes rapidly and efficiently at a constant temperature (37–42 °C) in 20–40 min [30,31,32,33,34,35,36]. This assay is based on using the recombinase enzyme and accessory proteins for DNA amplification and denaturation [37]. The species-specific primers are attached to the template DNA and amplify the DNA with the aid of DNA polymerase. The resulting product can be visualized by agarose gel electrophoresis, fluorescence produced by SYBR Green I nucleic acid, lateral flow assay, or even in real time using a fluorescent probe [30]. In a previous study, an RPA assay for the identification of individual nematodes of *P. allius* species was developed [38]. The developed assay could detect *P. allius* individuals that were obtained after processing the soil using nematode extraction methods. Although the developed RPA assay has advantages in terms of constant temperature settings and relatively less time for amplification, the assay was incapable of detecting *P. allius* nematodes directly from the soil without conducting nematode extraction. The RPA assay reported was also dependent on an agarose gel electrophoresis and SYBR Green I nucleic acid stain for visualization. DNA amplification by RPA can also be detected in real time using a probe that has a fluorophore, tetrahydrofuran, and quencher with a blocked spacer end [37]. The fluorophore probe binds to the amplified DNA strand within the RPA primers. This allows exonuclease to cleave the tetrahydrofuran attached to the probe, leading to 3′-end opening and hence DNA extension, which generally occurs in less than 10 min [37]. Real-time RPA has been referred to as having an advantage over the other modes of RPA product visualization as it is simple, portable, and requires significantly less time for detection [39].

This study was mainly aimed at developing a molecular diagnostic technique with the goal of reducing the time of *P. allius* detection in infested field samples. The specific objective of this study was to develop a real-time RPA assay that can rapidly detect the presence of *P. allius* in DNA extracted from soil using a commercial kit without adopting the traditional nematode extraction process. In this study, a fluorophore-labeled probe that produces fluorescent signals using a portable fluorometer was developed. Naturally infested field soil samples with *P. allius* collected from North Dakota were used to develop and verify the real-time RPA assay to detect *P. allius* DNA directly from the soil. To the best of our knowledge, this is the first report of real-time RPA developed for *P. allius* detection directly from soil DNA extracts.

## 2. Results

### 2.1. RPA Specificity

Evaluation of the specificity of the real-time RPA ensured that the primers and the designed probe with a fluorophore attached (Table 1) exclusively detected *P. allius* collected from three different states (North Dakota, Idaho, and Washington) and that there were no false positives for other nematodes (Table 2). It was observed that a very clear positive signal in terms of a high rise in fluorescence intensity was associated with the DNA of *P. allius,* and there was no rise in fluorescence intensity for the two non-template controls using sterilized double-distilled water instead of DNA (Supplementary Figure S1). The other non-target plant-parasitic nematodes from North Dakota, such as *Helicotylenchus* sp., *Heterodera glycines, Hoplolaimus* sp., *Pratylenchus dakotaensis*, *P. scribneri P. neglectus*, *Paratylenchus* sp., *Tylenchorhynchus* sp., and *Xiphinema americanum,* did not show a rise in fluorescence intensity beyond the threshold line (Table 2, Supplementary Figure S1). No positive signals for DNA of other stubby root nematode species, including *Trichodorus obtusus* from Florida, *T. obtusus* from South Carolina, *Paratrichodorus porosus* from South Carolina, and *Nanidorus minor* from North Carolina, were observed (Table 2). Similarly, no false positives for free-living and nematode communities were observed using RPA (Table 2). A positive control of the DNA of four nematodes, previously detected as *P. allius* DNA by qPCR, was used in the reaction to ensure that the RPA reaction was successfully conducted. The experiment, repeated three times with biological replicates, confirmed the specificity of the RPA assay.

### 2.2. RPA Sensitivity

#### 2.2.1. Real-Time Detection of DNA Extracted from Nematode Individuals

**Figure 1:**
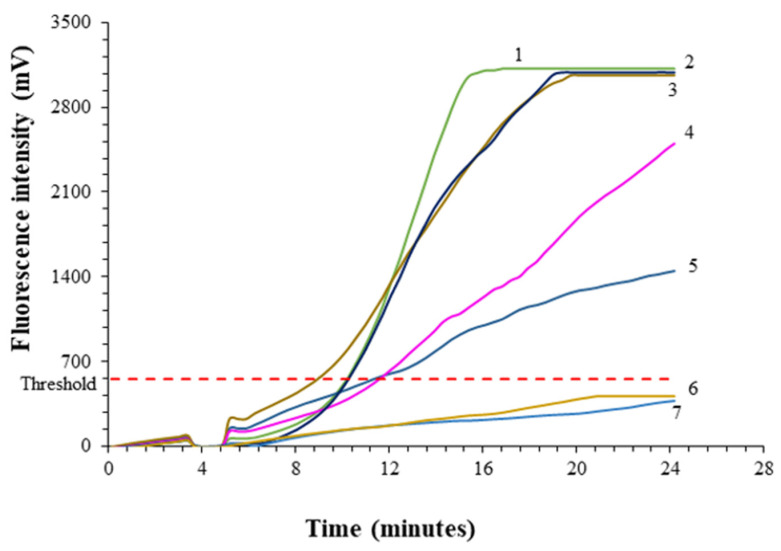
Real-time amplification plot of recombinase polymerase amplification (RPA) assay runs of serially diluted DNA extracts of *P. allius* for determining the sensitivity of RPA. 1 = Positive control (previously confirmed *P. allius* DNA of 4 nematodes using species-specific PCR), 3 = 1 nematode DNA, 2 = serially diluted DNA equivalent to 1/2 nematode, 4 = serially diluted DNA equivalent to 1/4 nematode, 5 = serially diluted DNA equivalent to 1/8 nematode, 6 = serially diluted DNA equivalent to 1/16 nematode, 7 = NTC (non-template control using sterilized double-distilled water instead of DNA). DNA was extracted from a single nematode using the Proteinase K method. The RPA amplification assay was carried out using exo kit reagents, species-specific RPA primers (MG2F/MG2R), and FAM-labeled probe (Pro 13). The threshold (red-dotted line) was computed based on the fluorescence intensity of eight non-template controls. Threshold = Average (NTC) + 4.785 × Standard deviation (NTC). The multiplication factor 4.785 corresponds to the 99.9% confidence interval of the t-distribution with seven degrees of freedom [42]. This high confidence interval was chosen to strengthen the specificity of the assay.

Since this study represents the first development of real-time RPA, it needs to be tested using *P. allius* specimens. The lowest limit of detection using the real-time RPA assay was evaluated with DNA extracted from nematode individuals. The fluorescence intensity of the amplified samples diminished with the reduction in DNA concentration. For RPA reaction with a DNA template of four nematodes (positive control), the fluorescent signals showed a higher peak compared to single nematodes as well as two-fold dilutions of the nematode DNA. Real-time RPA detected DNA equivalent to a one-eighth portion of a single nematode, but a one-sixteenth portion of a nematode’s DNA was not able to cross the threshold line of real-time RPA. There were no positive signals for the non-template control using double-distilled water instead of DNA (Figure 1).

#### 2.2.2. Real-Time Detection of DNA Extracted Directly from Artificially Infested Soil

**Figure 2:**
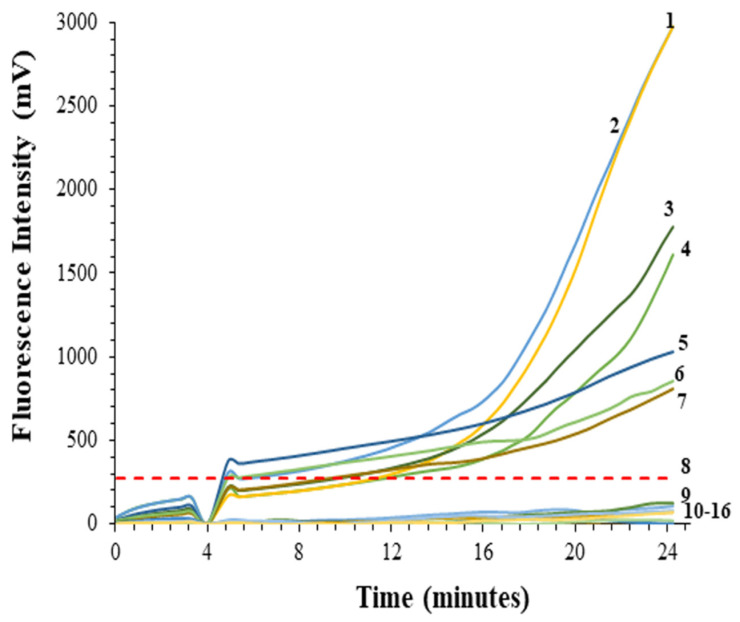
Recombinase polymerase amplification (RPA) of DNA extracted from soil samples artificially inoculated with different numbers of *Paratrichodorus allius*. Autoclaved soils inoculated with 4, 2, and 1 specimens of *P. allius* were used for DNA extraction. Soil DNA extracts were serially diluted two-fold and used to conduct the RPA assay. 1 = previously identified DNA with 4 *P. allius* specimens (positive control), 2 = DNA from soil equivalent to 2 *P. allius* specimens’ DNA, 3 = DNA from soil equivalent to 1 nematode’s DNA, 4 = serially diluted DNA from soil equivalent to ½ a nematode, 5 = serially diluted DNA from soil equivalent to ¼ of a nematode, 6 = serially diluted DNA from soil equivalent to 1/8 of a nematode, 7 = serially diluted DNA from soil equivalent to 1/16 of a nematode, 8 = threshold, 9 = serially diluted DNA from soil equivalent to 1/32 of a nematode, 10–16 = 7 non-template controls using DNA extracted from autoclaved soil without inoculation. The threshold was computed based on the fluorescence intensity of seven non-template controls. Threshold = Average (NTC) + 5.208 × Standard deviation (NTC). The multiplication factor 5.208 corresponds to the 99.9% confidence interval of the t-distribution with six degrees of freedom [42]. This high confidence interval was chosen to strengthen the specificity of the assay.

DNA extracted from autoclaved soil artificially inoculated with different numbers of *P. allius* (four, two, and one) demonstrated the RPA assay’s sensitivity. The RPA assay successfully amplified the DNA from artificially infested soil, as indicated by the rise in fluorescence intensity. The autoclaved soil with no inoculation did not show any rise in fluorescence intensity (Figure 2). The rise in fluorescence intensity varied with the number of nematodes present. The fluorescence intensity was higher for soil inoculated with four nematodes, lower for two nematodes per soil sample, and relatively low for single nematodes. Even with single nematode inoculation, a noticeable rise in fluorescence intensity was observed (Figure 2). The real-time RPA assay was able to detect as low as one-sixteenth portion of a single nematode in DNA extracted directly from soil (Figure 2). This showed that the sensitivity of the RPA assay was higher for soil than the limit of detection of RPA for nematode individuals.

### 2.3. Regression Analysis of Threshold Time and Nematode Number

**Figure 3:**
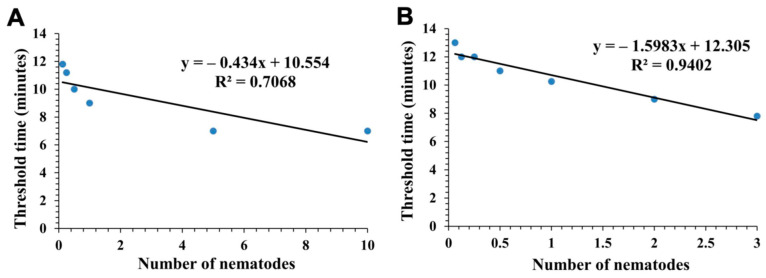
Threshold time estimation of *Paratrichodorus allius* detection using real-time RPA assay. The graph represents the time taken to detect DNA extracts from different numbers of *P. allius* specimens using real-time RPA. Data represent the average results of three replicates. Linear regression was observed between the concentration of DNA of *P. allius* and the threshold time required by the RPA assay to detect it. (**A**) Comparison between the concentration of DNA extracted from the nematode individuals using Proteinase K and the threshold time. (**B**) Comparison between the concentration of DNA extracted from soil inoculated with different numbers of nematodes and the threshold time. RPA was conducted using three replicates in both (**A**,**B**). Threshold time refers to the time when the fluorescent signal of the DNA sample in RPA crosses the threshold line and shows a positive signal, indicating the presence of the target nematode, *P. allius*.

The regression analysis showed a linear negative relationship between the number of *P. allius* individuals present in a DNA sample and the threshold time of RPA for positive signals. As the number of nematodes increased in a sample, the threshold time for RPA to detect *P. allius* decreased (Figure 3). The real-time RPA assay was able to detect the presence of *P. allius* in 7–14 min in all the samples where DNA was extracted either using the Proteinase K method (Figure 3A) or directly from soil (Figure 3B).

### 2.4. Direct Detection of P. allius from Naturally Infested Field Soil

Field soil samples collected from Sargent County, ND, infested with *P. allius*, validated the RPA assay. RPA reactions confirmed the presence of *P. allius* in 13 out of 15 soil samples (Table 3). *Paratrichodorus allius* DNA was verified using the species-specific qPCR assay [23], which provided similar detection as RPA. In addition, soil samples from the 15 fields were processed with traditional methods [43] and nematode densities were assessed by counting *P. allius* specimens in water suspensions using a microscope. When the nematode densities in these 15 fields were compared with the results of RPA detection of *P. allius* expressed as fluorescence intensity, there was a match between nematode densities and the intensity of fluorescence in 14 out of 15 samples (Table 3). Based on our results, we found that RPA could detect as few as 150 nematodes per kg of soil.

## 3. Discussion

In this study, real-time RPA was developed to rapidly detect *P. allius* directly in DNA extracts from field soils, without going through nematode extraction methods (such as the sugar centrifugal floatation method). The developed RPA assay has hastened the *P. allius* detection process since the results were visible simultaneously along with the amplification of the DNA via the portable isothermal machine (T16-ISO).

The developed RPA assay was specific to *P. allius* and demonstrated high specificity similar to previously developed molecular techniques such as conventional PCR and qPCR assays [23]. In the RPA assay, a sharp rise in fluorescence intensity was observed for only *P. allius*, while fluorescence remained below the threshold for other non-target DNA samples. The species-specific probe developed in this study exhibited higher specificity not only at the genus level but also enabled differentiation of *P. allius* from other stubby root nematode species tested. Because of the highly specific probe design, the positive fluorescent signals observed were exclusive to *P. allius*, which reduced the chances of false positives due to species that are closely related to *P. allius,* which could be a problem, especially in the case of non-specific binding of SYBR Green dye.

Setting a threshold value for real-time RPA analyses is challenging because the reaction is chemical rather than thermal-dependent [44]. The reaction initiates as soon as magnesium acetate is added to the RPA reagent mix and does not require a high denaturation temperature of 94 °C (as in the case of PCR) to start amplification. As a result, every sample in the same eight-tube strip of real-time RPA assay showed a slight difference in the initiation point of reaction. To ensure effective detection during the RPA assay, a threshold was set based on the formula used by Cherkaoui et al. [45]. Based on the current study, we found that a single threshold value cannot be used for all RPA reactions. Unlike PCR, which operates at a particular annealing temperature, RPA annealing is not regulated by any particular temperature. The annealing for RPA can occur even at ambient temperature [44] and it is not possible to control the initiation of the RPA reaction. The positive samples with a single nematode DNA extract showed a significantly high amplification curve (Figure 2), and thus might not need threshold calculations for detection purposes, while for accurate detection of *P. allius* DNA extracts with less than a single nematode, a separate threshold could be calculated for every run using water as a negative control to see the initiation reaction point and to separate it from background fluorescence.

The real-time RPA not only bypasses the initial steps of nematode extraction (e.g., sugar centrifugal floatation), but also eliminates the post-processing of the RPA product for visualizing the results, which were required for detecting *P. allius* in the previously developed agarose gel-based RPA assay as well as the SYBR Green I-based RPA assay [38]. In the agarose gel-based RPA method, purification of the RPA product was required, and result visualization took 75 min [38], while SYBR Green I-based RPA did not require purification and results were visualized after the RPA reaction, requiring 20 min for completion. Agarose gel and SYBR Green I-based RPA assays were reported to detect *P. allius* from nematode individuals, but not directly from the soil DNA [38]. Also, real-time PCR has not been previously reported for detecting *P. allius* from nematode individuals. For real-time RPA, no purification of the RPA product is required, and the amplified DNA is visualized simultaneously, via fluorescent signals produced by the probe, on a computer screen attached to a fluorometer (T 16-ISO), shortening the time to visualization of the results. Therefore, the real-time RPA assay could be a good alternative for the detection of *P. allius* from nematode individuals as well as direct detection in DNA extracts from field soils.

On the contrary, during the extraction of DNA directly from the soil, some inhibitory substances present in the soil, such as humic acid, are also co-extracted [46]. These inhibitors can hamper amplification of the DNA in qPCR and PCR [46], requiring qPCR modifications with either some chemicals or dilution of DNA to overcome the inhibitor’s effect. The real-time RPA assay showed tolerance to inhibitors [37]. The results of our study also suggest that DNA extracted directly from soil (artificially inoculated with *P. allius* nematodes) using the QIAGEN kit could be amplified by a real-time RPA reaction without the need for additional use of any reagents to resist inhibitors. Huang et al. [25] mentioned the addition of bovine serum albumin (BSA) for amplifying soil DNA, whereas in our current study using real-time RPA, DNA amplification was achieved without the addition of BSA. Previously developed RPA assays using agarose gel electrophoresis and SYBR Green I dye were unable to amplify DNA extracted directly from soil [38]. This could be due to the ineffectiveness of the TwistDx basic kit, which was used for the RPA–agarose gel electrophoresis and RPA–SYBR Green I dye assays for amplifying DNA extracted from soil. In the real-time RPA assay, the TwistDx exo kit was used and all the soil samples artificially inoculated with *P. allius* showed positive results.

Based on our results, the real-time RPA assay demonstrated greater sensitivity than other modes of RPA product visualization. The agarose gel-based RPA could detect up to one-quarter of a single nematode and the SYBR Green I assay was limited to single nematode DNA detection only, while the developed real-time RPA assay was able to detect one-eighth of a single nematode DNA template extracted from nematode individuals in a 50 µL RPA reaction. In our study, real-time RPA exhibited higher sensitivity for detecting DNA directly from soil compared to nematode individuals. It was capable of detecting one-sixteenth of a single nematode, which is equivalent to 0.75 ng of DNA per 50 µL RPA reaction. The real-time RPA assay for nematode individuals was less sensitive than for artificially infested soils, possibly due to differences in DNA extraction methods. The detection limit of real-time RPA for *P. allius* is lower than that of the developed PCR and qPCR molecular techniques, with conventional PCR capable of detecting DNA equivalent to 1/3125th of a nematode (equivalent to 0.00096 ng of DNA) and qPCR capable of detecting DNA equivalent to 1/15,625th of a nematode (equivalent to 0.000192 ng of DNA) [23,38]. The lower sensitivity of RPA could be due to variations in the initial DNA template concentrations before dilutions [36]. In some cases, the RPA assay shows higher sensitivity than PCR, such as in the detection of stem nematodes in sweet potatoes [47]. The higher sensitivity of RPA also depends on the efficiency of DNA extraction methods [6].

The specificity of the RPA assay was validated in soil samples naturally infested with *P. allius* by testing DNA extracted directly from the soil using species-specific qPCR. Nematode densities recorded by microscopic analysis and nematode detection by RPA assay were in agreement, confirming the effectiveness of the assay. Two of the three samples with low *P. allius* density (ND-9 and ND-15, as mentioned in Table 2) were not detected with RPA, suggesting that RPA may not be stable for samples with low densities but works well for medium and high-density *P. allius* soil samples. In the current study, we found that RPA could detect *P. allius* at nematode densities of 150 specimens/kg of soil. Some variations were observed among the biological replicates of the same soil sample due to possible non-uniform nematode distribution. It is worth mentioning that the chances of detecting nematodes were much higher in traditional soil samples, consisting of 200 g of soil that was used to microscopically assess nematode densities, than in only 0.5 g of soil that was used for extracting DNA for the RPA assay [48]. Variability in nematode densities among the replicates of small soil samples is common and has been reported for *Meloidogyne javanica*, *Pratylenchus zeae*, *Xiphinema elongatum*, and *P. neglectus* [49,50]. A higher number of replications in future experiments could possibly enhance the accuracy of the detection of DNA directly extracted from soil.

Since real-time RPA can be performed on a portable instrument, T16-ISO, and requires less time and a lower constant temperature, this assay has eased the detection of *P. allius*. DNA extraction directly from the soil sample accelerated the nematode detection process. The developed assay does not require knowledge of morphological characters of *P. allius* for species identification, hence making its detection very easy for diagnostic laboratories and plant clinics. Although the developed real-time RPA assay has increased DNA amplification and visualization of RPA products, it has a limitation in the preparation of soil DNA extracts, which at present, cannot be carried out directly on-site. In the future, with improved methods of DNA extraction from soil, this assay could be applied to on-site field detection of *P. allius*. Different methods for extracting *P. allius* DNA directly from soil could be explored, which would allow the assay to be applied on-site.

In this study, real-time RPA was developed to rapidly detect *P. allius*. The developed assay can be performed easily with a portable instrument (T16-ISO), primers, a probe, and RPA kit reagents. In conclusion, we have developed a simple, highly specific, and rapid molecular diagnostic technique, the real-time RPA assay, for the detection of *P. allius* directly from soil DNA extracts using a portable device.

## 4. Materials and Methods

### 4.1. Nematode Collection

Plant-parasitic nematodes were obtained from various sources (Table 2). *P. allius* nematodes were recovered from soil samples of North Dakota, Idaho, and Washington. Other plant-parasitic nematodes, including dagger nematodes, pin nematodes, ring nematodes, root-lesion nematodes, spiral nematodes, stunt nematodes, and a population of soybean cyst nematodes, were extracted from different field soil samples from North Dakota.

Briefly, the nematode samples with codes 1 and 13–27 (Table 2) were collected during the current research from North Dakota field soils. Soil samples with codes 2 and 3 were obtained from *P. allius*-infested field samples from Idaho and Washington, respectively [38]. Sample codes 4–12 represent DNA samples of non-target species of stubby root nematodes from different regions of the United States and were acquired from a previous study conducted by our lab [23]. Sample codes 22–24 were free-living/non-plant-parasitic nematodes, while soil samples with codes 25–27 consisted of nematode communities in the soil other than stubby root nematodes (Table 2). These non-plant-parasitic nematodes and other nematode communities were randomly collected from different field locations in North Dakota.

### 4.2. Nematode Extraction from Soil and Genus Identification

Nematodes from soil samples (Table 2) were processed for nematode extraction followed by nematode identification at the morphological level. The nematodes were extracted using the sugar centrifugal floatation method [43]. In this method, 200 g of sub-samples from each soil were taken and exposed to decanting and sieving, followed by centrifugation at 4000 rpm for 10 min. Finally, a sugar solution (1.3 M) was added to the soil to extract the nematodes out of the soil into the supernatant, and the nematode suspension was collected into a 50 mL vial. To identify the nematodes at the genus level, key morphological characteristics were observed using an inverted transmitted light microscope (Zeiss, Primovert lab microscope, Zeiss, Thornwood, NY, USA) [40]. All the non-target plant-parasitic nematodes identified were collected for DNA extraction and used as control nematode species for development of the real-time RPA assay.

### 4.3. DNA Extraction

DNA was extracted from the nematode individuals as well as directly from the soil. For nematode individuals, DNA extraction was performed using the Proteinase K method [28]. Nematodes identified under the microscope were picked using a dental pick and placed on the glass microscope slide with 10 µL of sterilized double-distilled water. Using the same dental pick, the nematode was bisected. The nematode pieces in the double-distilled water were pipetted and transferred to a 0.5 mL microcentrifuge tube. In the microcentrifuge tube, 2 µL of Proteinase K (600 µg/mL), 2 µL of buffer (10 × PCR buffer), and 6 µL of double-distilled water were added. After a gentle vortex and spinning, the microcentrifuge tubes were incubated at −20 °C for an hour, at 65 °C for an hour, and then at 95 °C for 10–15 min. The resulting nematode DNA was ready for use or could be stored at −20 °C.

DNA extraction directly from soil was conducted using the QIAGEN Power Soil DNA isolation kit (MoBio Laboratories, Inc., Carlsbad, CA, USA). The field soil samples were sub-sampled, and 15 g of soil from each sample was crushed in a pestle and mortar (70 mm in height and 90 mm in diameter), and the soil was dried overnight. DNA was extracted from the dried soil as per the manufacturer’s instructions, with a slight modification: instead of 0.25 g of soil sample as stated by the manufacturer, 0.5 g of soil was taken for each sample [49]. For creating artificially infested soil, the field soil was first autoclaved at 121 °C and a pressure of 115 kPa for 45 min. The autoclaved soil samples (0.5 g each) were then inoculated artificially with a variable number of *P. allius* (4, 2, and 1), and DNA was extracted following the same procedure as mentioned above, where the soil was crushed and DNA was extracted using the QIAGEN Power Soil DNA isolation kit.

For each DNA extraction, three independent biological replicates of every sample were used. The extracted DNA samples from both methods were then stored at −20 °C for further use.

### 4.4. Species Confirmation

The DNA of *Paratrichodorus* nematodes was amplified by the species-specific conventional PCR assay for *P. allius* using primers PaF11/PaR12 [23] (Table 2). For the DNA extracted from soil, species-specific qPCR assay [25] was performed to detect the presence of *P. allius* (Table 2).

### 4.5. RPA Probe Design

A 56 bp oligonucleotide, referred to as a probe, was designed as stated by the manufacturer (TwistDx Limited, Maidenhead, UK). The designed probe was homologous to the target sequence, with forward and reverse primers flanking the ends of the target sequence. To design a specific probe for *P. allius*, the sequences of *P. allius* (GenBank accession numbers KU094059, AM087124, KJ934124, and KT892735) were aligned with those of seven isolates of other *Paratrichodorous* spp. (JN123376, AY430187, MG739667, KJ934126, GU645907, JQ217080, and AM087125) using ClustalW (a multi-alignment tool) of BioEdit (Version 7.2) [51]. The specificity of the probe was further examined by the NCBI BLAST tool (https://blast.ncbi.nlm.nih.gov/Blast.cgi) (accessed on 25 November 2021) [52]. The probe (Pro 13) showed high similarity (100%) only to the sequences of the ITS1 rDNA region of *P. allius* isolates. The designed probe sequence was further tested to avoid the self-dimers or secondary structures using OligoCalc (http://biotools.nubic.northwestern.edu/OligoCalc.html), an online calculator [53], and the Multiple Primer Analyser web tool (https://www.thermofisher.com/us/en/home/brands/thermo-scientific/molecular-biology/molecular-biology-learning-center/molecular-biology-resource-library/thermo-scientific-web-tools/multiple-primer-analyzer.html) (accessed on 27 November 2021). The probe was synthesized by Biosearch Technologies, Inc. (Petaluma, CA, USA). To produce fluorescent signals, the synthesized probe consists of a fluorophore (FAM), an internal abasic nucleotide analog, a tetrahydrofuran (THF) residue incorporated within the probe, and a polymerase extension blocking group (C-3 spacer) attached to the 3′ end (Table 1).

### 4.6. Development of Real-Time RPA Assay

The real-time RPA assay was conducted using the FAM-labeled probe, MG2F/MG2R primers [38], and commercially available TwistAmp exo kit reagents (TwistDx Limited, Maidenhead, UK) for DNA amplification in a fluorometer. To obtain the optimal temperature for the RPA reaction, different temperatures were tested. For the RPA reaction, the manufacturer’s instructions, as given in the TwistAmp exo kit manual, were followed but with slight modifications. A master mix of 29.5 µL of dehydrating buffer was added to 2.4 µL of each forward and reverse primer, (MG2F/MG2R, 10 µM each), 0.9 µL of probe (Pro 13), and 10.3 µL of sterilized double-distilled water. This master mix was added to the dried reagents that contain the enzymatic mixture (provided in the kit). A 2 µL volume of *P. allius* DNA was pipetted into the RPA reaction tube. Finally, 2.5 µL of 280 mM magnesium acetate was added to make a 50 µL volume of RPA reaction.

RPA reaction was carried out using the portable fluorescent reader T16-ISO instrument (Axxin, El Segundo, CA, USA). This instrument was attached to the laptop and the results were visualized simultaneously. The RPA reaction mixture was placed inside the T16-ISO, and different temperatures (37 °C, 38 °C, 39 °C, and 40 °C) were evaluated for 20 min. The most efficient results were obtained when the temperature was set to 40 °C, where the positive samples were observed in less than 20 min. The fluorescent reader, T16-ISO, records the fluorescent signals every 20 s. After the initial 4 min of the RPA reaction, the fluorescent reader was paused and the tubes were vortexed and spun 2–3 times, and then placed back in the T16-ISO machine in the same order as before.

### 4.7. Threshold Estimation for Real-Time RPA Assay

The RPA reaction was temperature sensitive and initiated as soon as magnesium acetate was added to the reaction [41]. This sensitivity led to a difference in background fluorescence among the simultaneously conducted RPA reactions. This background fluorescence was highly variable for RPA reactions in technical replicates performed in different environments. The setting of a threshold for T16-ISO was necessary to eliminate the variability. Additionally, while a clear rise in fluorescence intensity was observed for the DNA samples with single nematodes, detecting samples with lower DNA concentrations also needed the establishment of a threshold value. A threshold was calculated separately for every run using non-template controls (NTC) to avoid false results. The threshold was calculated using the following formula [45]:

Threshold = average (NTC) + 4.785 × standard deviation (NTC)

The value 4.785 refers to the 99% confidence interval of the t-distribution with seven degrees of freedom [45]. This value can vary depending on the number of non-template controls used in the reaction. The threshold estimation increases the analytical sensitivity of the RPA assay, and the high confidence interval used in the formula increases the analytical specificity of the assay.

The time at which the fluorescent signal crossed the threshold line was referred to as the threshold time. The relationship between nematode DNA (or DNA equivalent to different numbers of nematodes) and threshold time for detecting *P. allius* using RPA was investigated using regression analysis. Both DNA extracted from nematode individuals as well as DNA extracted directly from soil were included in the analysis. Each sample was replicated three times. Different numbers of nematodes in a sample were tested using real-time RPA and the time of detection was recorded. A graph was plotted based on the average threshold time of three replicates of each DNA sample.

### 4.8. Evaluation of RPA Specificity

Specificity testing was conducted using a combination of the primers MG2F/MG2R and the designed probe, Pro13, to confirm whether the developed real-time RPA assay was specific to *P. allius* only. The specificity was evaluated using DNA of non-target plant-parasitic nematodes (Table 2; codes 13–21), other species of stubby root nematodes (Table 2; codes 4–12) from different parts of the USA, randomly selected non-plant-parasitic nematodes present in North Dakotan fields (Table 2; codes 22–24), and DNA extracted directly from soil communities of nematodes other than stubby root nematodes (Table 2; codes 25–27). Three biological replicates of each sample were taken and tested for RPA reactions. The presence of DNA in all the samples was confirmed by PCR using the universal primers D2A/D3B [54]. All the DNA samples were further tested with previously published *P. allius*-specific primers, PaF11/PaR12, in conventional PCR and qPCR [23,25] for validating the specificity results of the RPA assay.

### 4.9. Evaluation of RPA Sensitivity

#### 4.9.1. For *Paratrichodorus allius* Nematode Individuals

Real-time RPA was evaluated for sensitivity using nematode individuals. Nematode DNA was extracted using the Proteinase K method. The extracted DNA was then diluted by sequential two-fold serial dilutions of *P. allius*. For nematode individuals, one *P. allius* nematode per sample was extracted and diluted in the ratios 1:1, 1:2, 1:4, 1:8, 1:16, and 1:32, which are equivalent to 1, 1/2, 1/4, 1/8, 1/16, and 1/32 portions of a nematode individual. Three biological replicates of each nematode DNA were taken for amplification using real-time RPA. In each reaction, a positive control (comprising DNA of four *P. allius* nematodes) whose identity was previously confirmed using species-specific qPCR was used. Also, negative or non-template control (autoclaved water) was added to RPA reagents instead of template DNA to confirm that there was no contamination in the RPA reaction.

#### 4.9.2. For Direct Detection of *Paratrichodorus allius* from Artificially Infested Soil

The real-time RPA assay was evaluated for detecting *P. allius* directly from the soil. The autoclaved soil was artificially inoculated with different numbers of nematodes (4, 2, and 1). There were three replications of each artificially infested soil sample. Further, all the RPA reactions were carried out using three biological replicates of each soil sample. Every reaction consisted of a positive control, which contained the previously identified DNA of four *P. allius* nematodes. For a negative control, autoclaved soil without inoculation was also used, and DNA extraction followed by RPA reaction was performed.

DNA extracts from the single nematodes in the soil were then diluted by two-fold serial dilutions (DNA equivalent to 1, 1/2, 1/4, 1/8, 1/16, and 1/32). Three biological replicates of each sample were taken. Real-time RPA reaction was then carried out as stated above. The results were visualized as the reaction was running, within 20 min, using T16-ISO.

### 4.10. Validation of the Developed RPA Assay Using Field Soil Samples

Fifteen soil samples were collected from potato fields in Sargent County, ND (Table 3). DNA was extracted directly from each soil sample using the QIAGEN Power Soil DNA isolation kit following the manufacturer’s instructions. The real-time RPA assay was conducted to detect the presence of nematodes in the soil samples. Three replicates of each soil sample were used for DNA extraction. Simultaneously, the same soil samples were also used to extract nematodes using the traditional sugar centrifugal floatation method, resulting in nematode suspensions. The nematodes were counted manually under an inverted transmitted light microscope (Zeiss, Primovert lab microscope, Zeiss, Thornwood, NY, USA) using the counting slide (Chalex, LLC, Wallowa, OR, USA). From the nematode suspensions, a 1 mL aliquot was placed on the slide and the nematodes were identified based on morphological features, as explained earlier. The nematodes were counted in 1 mL of suspension and the population density was estimated for 200 g of a sub-sample of the soil, followed by extrapolation for 1 kg of soil using the binary method. The nematode population density determined by the traditional nematode extraction and microscopic counting was compared to the intensity of the fluorescent signals produced from the soil samples using the RPA assay.

## Figures and Tables

**Table 1:** Primers and probe used in the development of real-time RPA for detection of *P. allius* in DNA extracted from soil.

Primers	Sequences (5′→3′)	Length (bp)	Reference
D2A	ACAAGTACCGTGAGGGAAAGTTG	23	[40,41]
D3B	TCGGAAGGAACCAGCTACTA	20	
PaF11	AAGCTTGCTGGTAGTTTGTTGG	22	[23]
PaR12	AAGTAGTTAAAAGGGGAGTCG	21	
MG2F	TCGGTCCTAGCTGGCAGGCCATATTCATAAGC	32	[38]
MG2R	GCAGGTGTAACTGTAAGACCACACAGTCGACGT	33	
**Probe**			
Pro13	GAATGGGCTCAGAAGGTCCCTTTATGAGTGGCTT-T(FAM)-G-THF-G-T(BHQ-1)-GTCGCTTCTACACGCTT-Spacer C3	56	This study

FAM refers to fluorescein amidites, used as fluorophore dye; THF refers to tetrahydrofuran, used as an abasic nucleotide analog; BHQ-1 is a black hole quencher used as a quencher to absorb energy from a fluorophore; and Spacer C3 is a 3′–modification group for blocking the probe from polymerase extension.

**Table 2:** Nematode species used in the study for testing primer and probe specificity in the real-time RPA assay.

Code ^a^	Nematode Species	Origin	Sample ID	Crop ^b^	PCR ^c^	qPCR ^d^	RPA ^e^
1	*Paratrichodorus allius*	North Dakota	ND-1	Potato	+	26.35 ± 0.50	+
2	*P. allius*	Idaho	I-1	Potato	+	28.24 ± 0.40	+
3	*P. allius*	Washington	W-1	Potato	+	22.73 ± 0.49	+
4	*Nanidorus minor*	North Carolina	NC-34214	Soybean	-	N/A	-
5	*N. minor*	North Carolina	NC-34538	Soybean	-	N/A	-
6	*N. minor*	North Carolina	NC-34537	Soybean	-	N/A	-
7	*N. minor*	North Carolina	NC-34536	Soybean	-	N/A	-
8	*N. minor*	North Carolina	NC-34546	Soybean	-	N/A	-
9	*N. minor*	North Carolina	NC-32943	Soybean	-	N/A	-
10	*P. porosus*	South Carolina	SC-C	Spinach	-	N/A	-
11	*Trichodorus obtusus*	Florida	FL-1	Golfgrass	-	N/A	-
12	*T. obtusus*	South Carolina	SC-G	Golfgrass	-	N/A	-
13	*Helicotylenchus* sp.	North Dakota	Spi	Potato	-	N/A	-
14	*Heterodera glycines*	North Dakota	SCN	Soybean	-	N/A	-
15	*Hoplolaimus* sp.	North Dakota	Hop	Soybean	-	N/A	-
16	*Paratylenchus* sp.	North Dakota	Prt	Field pea	-	N/A	-
17	*Pratylenchus dakotaensis*	North Dakota	Hg50-1	Soybean	-	N/A	-
18	*P. neglectus*	North Dakota	Pn1	Corn	-	N/A	-
19	*P. scribneri*	North Dakota	Ps1	Corn	-	N/A	-
20	*Tylenchorhynchus* sp.	North Dakota	Tyl	Potato	-	N/A	-
21	*Xiphinema americanum*	North Dakota	Xph	Potato	-	N/A	-
22	Free-living nematode 1	North Dakota	NPN1	Potato	-	N/A	-
23	Free-living nematode 2	North Dakota	NPN2	Potato	-	N/A	-
24	Free-living nematode 3	North Dakota	NPN3	Potato	-	N/A	-
25	Nematode community 1	North Dakota	S-1	N/A	-	N/A	-
26	Nematode community 2	North Dakota	S-2	N/A	-	N/A	-
27	Nematode community 3	North Dakota	S-3	N/A	-	N/A	-

^a^ Samples 1–3 and 13–27 were collected in this research study. Samples 1–3 were collected from potato fields—sample 1 from North Dakota, sample 2 from Idaho, and sample 3 from Washington. Samples 4–12 are other stubby root nematode species from different states in the U.S. collected in previous research by our lab [23]. Samples 22–24 are non-parasitic nematodes randomly extracted from potato fields in North Dakota. Samples 25–27 are free-living nematodes and nematode communities 1, 2, and 3 that contain plant-parasitic nematodes other than stubby root nematodes in the soil. ^b^ Crops planted in the soils from where the soil samples were collected. ^c^ PCR was conducted to verify the specificity of the RPA assay using species-specific primers PaF11/PaR12 [23]. Symbols + or - indicate the presence or absence of a single amplicon, respectively, on the agarose gel electrophoresis of the PCR product. The agarose gel electrophoresis images were published in our previous work [38]. ^d^ qPCR was conducted with primers PaF11/PaR12 [23] to verify the specificity of the RPA assay. The Cq values are presented as the mean ± standard deviation of the three replicates used in qPCR assays. N/A represents no amplification detected by qPCR assays. ^e^ RPA represents the results in the form of fluorescence intensity above the threshold. Symbol + indicates the rise in fluorescence intensity, indicating the presence of *P. allius*, while the symbol - refers to no or slight rise in fluorescence intensity below the threshold line, indicating samples negative for *P. allius* presence. Three biological replicates were used for each sample. Real-time RPA was conducted using the primers MG2F/MG2R [38] and the probe Pro 13 (this study).

**Table 3:** Field soil samples tested for *Paratrichodorus allius* presence using real-time RPA and comparisons between densities recorded by microscopic analysis and RPA detections expressed as fluorescence intensity.

Field Samples ^a^	SRN/kg ^b^	Population Density of SRN ^c^	RPA ^d^	Mean Fluorescence Intensity (mV)e ± Standard Deviation ^e^
ND-1	750	High density	positive	1243.8 ± 383.2
ND-2	700	High density	positive	731.8 ± 142.0
ND-3	900	High density	positive	837.2 ± 14.6
ND-4	1050	High density	positive	763.4 ± 551.5
ND-5	300	Medium density	positive	1540.4 ± 40.2
ND-6	300	Medium density	positive	487.4 ± 509.4
ND-7	300	Medium density	positive	563.5 ± 463.6
ND-8	300	Medium density	positive	1058.7 ± 56.8
ND-9	150	Low density	negative	141.1 ± 15.2
ND-10	150	Low density	positive	906.1 ± 871.7
ND-11	200	Medium density	positive	1164.8 ± 264.5
ND-12	150	Low density	positive	490 ± 203.9
ND-13	400	Medium density	positive	1545.1 ± 858.1
ND-14	600	High density	positive	2536.8 ± 1961.4
ND-15	0	Low density	negative	191.5 ± 122.5
16-control	0	Nil sample	negative	191.8 ± 54.9

^a^ Soil samples collected by the North Dakota State University Nematology lab from potato fields in Sargent County, North Dakota. ND-1 to ND-15 refer to the field soil samples and 16 refers to the autoclaved field soil sample used as a negative control. ^b^ The nematodes were extracted from soil samples using the sugar centrifugal floatation method. Stubby root nematodes (SRNs), *P. allius* in particular, were counted using a microscope. SRN density was calculated based on the number of SRNs per milliliter of nematode suspension extracted from 200 g of field soil. The number of SRNs/milliliter was then multiplied by 5 to obtain the number of SRNs/kg of soil. ^c^ Based on the SRN density observed in the data for field samples from a previously published paper on SRNs in North Dakota [25], the soil samples were categorized into high density (>500 stubby root nematodes/kg of soil), medium density (151–500 stubby root nematodes per kg of soil), and low density (0–150 stubby root nematodes per kg of soil). ^d,e^ DNA was extracted separately from 0.5 g of each soil sample, with three replicates of each soil sample, using the Power soil QIAGEN kit. The fluorescence intensity was calculated by subtracting the initial fluorescent signal at the start of the RPA reaction from the final fluorescent signal produced by each sample after 20 min. RPA was conducted three times for each soil sample. The fluorescent intensities of all the biological replicates of every soil sample were averaged. The threshold value (259.17) was calculated using the formula: Threshold = Average (NTC) + 318.31 × Standard deviation (NTC). The multiplication factor 318.31 corresponds to the 99.9% confidence interval of the t-distribution with one degree of freedom. NTC refers to the non-template control using water rather than DNA. All the values above this threshold were considered positive, and all those below this threshold were negative. The presence of *P. allius* was confirmed by performing a qPCR assay using published species-specific primers, PaF11/PaR12 [23].

## Data Availability

Data generated or analyzed during this study are included in this article.

## Supplementary Materials

The supporting information can be downloaded at: https://www.mdpi.com/article/10.3390/ijms251910371/s1.
