# Supplementary material for: Rapid and Direct Detection of the Stubby Root Nematode, Paratrichodorus allius, from Soil DNA Extracts Using Recombinase Polymerase Amplification Assay
Source: Int J Mol Sci. 2024 Sep 26;25(19):10371. doi: 10.3390/ijms251910371 (PMC11476371; doi:10.3390/ijms251910371)
Supplement: Supplementary file 1 [file ijms-25-10371-s001.zip › ijms-3132981-supplementary.pdf]

Supplementary materials

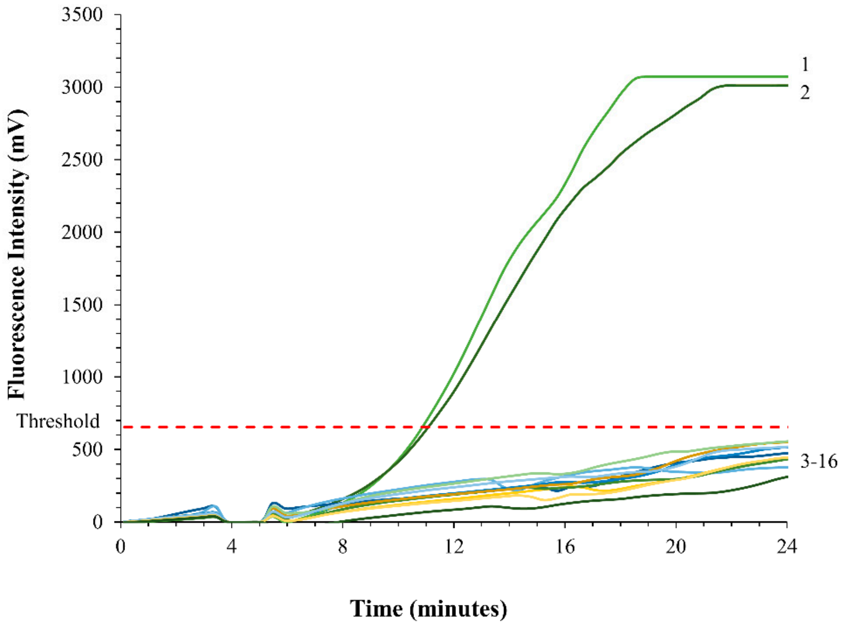

Figure S1. Real-time recombinase polymerase amplification (RPA) assay for testing specificity of primers (MG2F/MG2R) and probe (Pro 13) using confirmed nematode control species with TwistAmp® exo kit. 1 = Positive control (previously confirmed *Paratrichodorus allius* DNA of 4 nematodes using species-specific PCR), 2 = *P. allius* from North Dakota, 3 = *Trichodorus obtusus* from Florida, 4 = *T. obtusus* from South Carolina, 5 = *P. porosus* from South Carolina, 6 = *P. minor* from North Carolina, 7 = *Xiphinema americanum* from North Dakota, 8 = *Paratylenchus* sp. from North Dakota, 9 = *Helicotylenchus* sp. from North Dakota, 10 = *Hoplolaimus* sp. from North Dakota, 11 = *Pratylenchus scribneri* from North Dakota, 12 = *P. neglectus* from North Dakota, 13 = *Tylenchorhynchus* sp. from North Dakota, 14 = *Heterodera glycines* from North Dakota, 15, 16 = NTC (non-template control using water instead of DNA). The thresholds were computed based on fluorescence intensity of two negative controls (NTC). Threshold = Average (NTC) + 318.31 X Standard deviation (NTC). The multiplication factor 3.852 corresponds to the 99.9% confidence interval of the t-distribution with one degree of freedom. This high confidence interval was chosen to strengthen the specificity of the assay.
